# ‘Nothing About Us, Without Us’: Research Priorities for Autistic Girls, Women and Gender Diverse People in Australia

**DOI:** 10.1007/s10803-024-06330-5

**Published:** 2024-05-06

**Authors:** Rachel Grove, Hayley Clapham, Tess Moodie, Sarah Gurrin, Gabrielle Hall

**Affiliations:** School of Public Health, Faculty of Health, University of Technology Sydney, Ultimo, Australia

**Keywords:** Autism, Participatory research, Girls, Women, Gender diversity, Wellbeing, Health, Neurodivergence, Disability, Research priorities

## Abstract

Autistic girls, women and gender diverse people have specific needs that are underrepresented in research. Research priorities are often established by funding bodies, researchers, parents, carers and health professionals and may not meet the needs of the diverse Autistic community. This co-produced project aimed to identify what research would benefit the lives of Autistic girls, women and gender diverse people in Australia. We interviewed 47 Autistic girls, women and gender diverse people aged seven and above and obtained feedback from an additional 411 Autistic people through an online survey. Autistic young people identified six key research priorities including (1) better understanding and support at school, (2) understanding our experiences, strengths and challenges, (3) autism specific mental health support, (4) Autistic friendships and relationships, (5) experiences of gender diversity and (6) accommodations to make life easier for us. Eight key research priority areas were identified by Autistic adults including (1) understanding and supporting specific needs in adulthood, (2) experiences of trauma, abuse and sexual violence, (3) supporting mental health and wellbeing, (4) addressing barriers in healthcare, (5) understanding and supporting physical health needs, (6) addressing barriers in education and the workplace, (7) understanding the role of society, embracing neurodiversity and the importance of Autistic identity and (8) co-designing research and supports with Autistic people. We provide a discussion around the importance of focusing on these research priority areas in future autism research in Australia.

## Community Summary

This study was led by Autistic people. It aimed to develop research areas that are important to Autistic girls, women and gender diverse people aged seven and above in Australia. Autistic people aged between 7 and 17 identified six research areas they thought were important. These included being understood and supported at school, understanding their strengths and challenges, receiving mental health support, Autistic friendships and relationships, experiences of gender diversity and accommodations to make life easier for Autistic young people. Eight research areas were identified by Autistic adults, including understanding and supporting their needs in adulthood, experiences of trauma, abuse and sexual violence, supporting mental health and wellbeing, addressing barriers in healthcare, education and the workplace, understanding and supporting physical health needs, understanding the role of society, embracing neurodiversity and Autistic identity and co-designing research and supports with Autistic people.

## Introduction

Autistic people are calling for research that is driven by the Autistic community, aligned with the disability rights principle of ‘nothing about us without us’. Despite this, research priorities have historically been dictated by funding bodies or the non-autistic research community, and often do not correspond with the needs of Autistic people (Pellicano et al., 2014). A recent study outlined that, similar to the UK, Canada and the US, the majority of funding in Australia is allocated to biological research, rather than to topics that have been identified as important to the Autistic and autism communities^1^, such as supports and services (den Houting & Pellicano, 2019). In addition, priorities for autism research have often been set by parents, carers, health professionals and researchers. While there have been some recent attempts to engage the Autistic and autism communities in developing research priority areas in the US (Frazier et al., 2018), the UK (Cusack & Sterry, 2016; Pellicano et al., 2014), New Zealand (Emerson et al., 2023) and Australia (Australian Autism Research Council, 2019; Clark et al., 2022; Gatfield et al., 2016), Autistic people make up a small minority of the samples included in this research. This ranges from 7% in the UK, 9% in the US, to between 20% and 40% in some Australian and New Zealand studies. The number of Autistic people in this research has increased over time, with the most recent update by the Australasian Autism Research Council (AARC) conducting focus groups in which 60% of the participants were Autistic adults (AARC, 2021). However, there is no research to date that has developed research priorities based solely on the perspectives of Autistic adults (Chown et al., 2023). In addition, the needs of Autistic young people are often represented by proxies such as parents, carers or health professionals. While these are valuable perspectives, we need to empower young Autistic people to determine what research they think will benefit their lives.

Autistic people with intersectional identities also have unique experiences and specific needs that should be identified, addressed and prioritised in research. Autistic girls and women experience higher rates of mental and physical health conditions (Kassee et al., 2020; Tint et al., 2023), sexual violence (Cazalis et al., 2022) and have specific sexual and reproductive health needs (Graham Holmes et al., 2022). There is also evidence that Autistic women report higher rates of emergency department visits, hospitalisations, family doctor and neurologist visits than Autistic men (Tint et al., 2023), despite experiencing additional barriers to accessing these services. Almost 80% of the Autistic people who completed the AARC 2019 consultation on autism research priorities identified the need for research focused on women and girls, highlighting the need for more research in this area. Trans and gender diverse Autistic people also experience additional health inequities, including greater disparities in mental and physical health conditions (Wallisch et al., 2023), more unmet health needs (Wallisch et al., 2023) and a lack of access to gender-affirming care (Bruce et al., 2023; Strauss et al., 2021). There is a significant lack of research into the needs of Autistic gender diverse people, with research tending to focus on prevalence rates rather than on their research and support needs. It is imperative to understand the nature and impact of the inequity experienced by Autistic gender diverse people, and include their experiences, needs and priorities in research (Gratton et al., 2023).

Autistic academics are calling for the inclusion of Autistic people as partners in research, rather than just as research participants (Chown et al., 2017; den Houting, 2019). Participatory research engages, consults, co-produces and supports community-led and community controlled research with the Autistic community (den Houting, 2021). In this project, we used participatory research principles to co-produce research priorities for Autistic girls, women and gender diverse people in Australia. Co-production involves researchers and community members working together as equal partners from the beginning of the research process to develop a research question, design and implement a research project (den Houting, 2021). It is also critical to ensure that power and control over the project is shared by both researchers and community members (den Houting, 2021). This co-produced project asked Autistic girls, women and gender diverse people aged seven and above about what research would benefit their lives. This included cisgender girls and women, transgender, non-binary, gender diverse people and anyone who was socialised or identified as a woman or girl. We used the term gender diverse within this project to fit with the recommendations outlined by the United Nations (United Nations Office of the High Commissioner for Human Rights, 2023) and the Victorian Government (2021) in Australia. We acknowledge that language in this space is dynamic and are always open to feedback from the community on what language is appropriate and inclusive. This project aimed to identify separate research priorities for Autistic adults and Autistic young people, to account for their unique perspectives and needs.

## Methods

### Community Involvement

This project was led by a group of Autistic women (HC, SG, GH) and gender diverse (TM) adults working with a non-autistic researcher (RG). Regular meetings with all members of the research team occurred prior to the initial stages of the research, to develop the research questions, methodology and funding proposal. All Autistic members of the research team were paid for their time and an Autistic research assistant (GH) was employed to conduct the interviews. During the data collection phase, regular meetings occurred between GH and RG, with the broader team meeting frequently to discuss the transcripts generated as part of the qualitative data collection phase, leading to the development of the draft themes and subthemes. The research team then developed the online questionnaire to be utilised in stage two of the project, assisted with recruitment and advertising the project, and met frequently to discuss and develop the final set of research priorities. RG led the writing of the manuscript, with all members providing significant input into the quotes selected for inclusion. All authors made a significant contribution to the final manuscript. The whole research team were also involved in the dissemination of the research priorities through the development of a video, webpage and webinar (https://www.uts.edu.au/autistic-women-and-girls-research-priorities). These participatory methods were used to ensure that this research was informed by lived experience, to ensure that the questions and results were interpreted from an Autistic perspective and are meaningful and relevant to the Autistic community.

### Procedure

The project was conducted in two stages. Semi-structured interviews were completed, followed by an online survey. Decisions to structure the project in this way were made by the Autistic members of the research team. This was to ensure that we were able to capture rich data through the qualitative interviews and develop research priorities from the experiences of Autistic girls, women and gender diverse people, rather than imposing a pre-determined set of priorities for feedback as part of an online survey. We also used this methodology to ensure that we were able to obtain this information directly from Autistic young people, rather than through a proxy. Recruitment for both stages of the project was completed online, with the project advertised on social media through peak autism and disability organisations across Australia. We included Autistic girls, women and gender diverse people aged seven and above, who were currently living in Australia. We included Autistic people with either a formal diagnosis or who self-identified as Autistic. This is due to the reported barriers to accessing a diagnosis, including costs, the complexity of the healthcare system, as well as stigma, and a lack of support from health professionals (Huang et al., 2020; Lewis, 2017). It was important that self-identifying Autistic people were included in this research to ensure that we did not preference the needs of Autistic people who are able to access a diagnosis. Cisgender Autistic men were excluded from the project.

#### Stage 1: Semi-Structured Interviews

Semi-structured interviews were completed with 47 Autistic girls, women and gender diverse people. All interviews were conducted by an Autistic woman (GH). Sensory and communication needs were supported throughout this process, and everyone was invited to complete the interview in the way that suited them best, including over text, email, phone, or via video, audio, or the chat function on zoom. Young people were invited to have a parent or carer with them at any stage of the interview. However, we aimed to hear from young people individually where possible, to gain their direct perspectives. During the interviews, we asked a series of open-ended questions related to diagnosis, self-identification, day to day experiences, support, and research topics. These open-ended questions were provided to the interviewees prior to their interview, so that they had time to reflect on, or prepare their answers. Interviews were video and audio recorded, and then transcribed verbatim. The transcribed interviews were sent to each interviewee, to provide them with the opportunity to amend or add additional information. The interviewees were also asked to complete a short online background questionnaire that asked for demographic information, as well as about co-occurring conditions, employment and education.

#### Stage 2: Online Survey

The second stage of the project obtained additional feedback from 411 Autistic girls, women and gender diverse people on the draft research priorities that were identified separately for adults and young people during stage one. Autistic girls and gender diverse young people aged 7 to 17 were asked to rate each of the six draft priority areas on a three-point scale from ‘very important’, ‘a little important’ to ‘not important’. They were then asked to choose the two most important subtopics under each research priority heading. Autistic women and gender diverse adults (>= 18 years) were asked to rate the eleven draft research priority areas from most (1) to least (11) important. They were then asked to select the top three subtopics under each priority area. An open text field also enabled Autistic adults to identify any other research priority areas that they felt were important. A short background questionnaire was completed to obtain demographic information, as well as details about co-occurring conditions, employment and education.

### Participants

#### Stage 1: Semi-Structured Interviews

We interviewed 16 Autistic girls and gender diverse young people aged between 8 and 17 (Mean = 12, SD = 3). All the Autistic young people we interviewed were formally diagnosed as Autistic, with an age of diagnosis between 4 and 14 years (Mean = 8, SD = 3). 19% of these Autistic young people identified as non-binary and 94% were from a White European background. All the Autistic young people we interviewed used spoken communication and 13% reported an intellectual disability. Co-occurring mental health conditions were reported by 75%, and 56% reported one or more physical health condition.

Interviews were completed with 31 Autistic women and gender diverse adults aged between 21 and 63 (Mean = 39, SD = 12). Age of formal identification ranged between 3 and 55 years (Mean = 33, SD = 13). 17% of the adults we interviewed self-identified as Autistic, with age of self-identification ranging from 21 to 49 years (Mean = 34, SD = 11). The majority (77%) of the Autistic adults we interviewed were from a White European background, with 3% identifying as Aboriginal or Torres Strait Islander. A large proportion (70%) were in paid employment, 63% had completed a university degree and all used spoken communication. 40% of the Autistic adults we interviewed were parents, 50% of whom had Autistic children. Physical health conditions were reported by 63%, with 83% reporting one or more co-occurring mental health condition.

#### Stage 2: Online Survey

The online survey was completed by 81 Autistic girls and gender diverse young people, aged between 7 and 17 (Mean = 12, SD = 3). Most had received a formal diagnosis between the ages of 3 and 17 (Mean = 10, SD = 4), with 6% self-identifying as Autistic (Range = 12 to 16 years, Mean = 14, SD = 2). 37% of the Autistic young people we surveyed identified as gender diverse, 1% reported a co-occurring intellectual disability and 5% were non-speaking. One or more co-occurring mental health conditions were reported by 82% of Autistic young people, with 32% reporting one or more physical health condition. A large proportion (72%) were from a White European background, with 5% identifying as Aboriginal or Torres Strait Islander.

330 Autistic women and gender diverse adults aged between 18 to 71 (Mean = 36, SD = 11) completed the online survey. Age of formal identification ranged between 3 and 69 years (Mean = 32, SD = 12), with 22% of the adults we surveyed self-identifying as Autistic (Range = 12 to 60; Mean = 35, SD = 11). 20% were gender diverse, 2% reported an intellectual disability and 1% were non-speaking. More than half (54%) had completed a university degree and 67% were currently employed. Of the 48% of Autistic parents who completed the survey, 59% also had Autistic children. Most of the Autistic adults we surveyed (86%) were from a White European background, with 3% Aboriginal and Torres Strait Islander peoples included in the data. Co-occurring mental health conditions were reported by 91%, with 51% reporting one or more co-occurring physical health condition.

### Data Analysis

The interview data was analysed using Braun and Clarke’s (2006, 2019) method for reflexive thematic analysis. This method was able to capture both semantic and latent meanings within the data, as well as provide both descriptive and interpretative accounts. The data was conceptualised within the social model of disability (Oliver, 1983) and analysed using a social ecological lens, which conceptualises health as impacted by individual, interpersonal, community, policy and societal factors (Bronfenbrenner, 1989; McLeroy et al., 1988). Themes were developed using an inductive, or bottom up, approach. This involved identifying shared patterns of meaning within the interview transcripts. The data analysis was informed by the backgrounds and experiences of the team members, including training in psychology (RG and SG), disability advocacy (HC, GH and TM), nursing (GH) and gender-based violence (TM). RG listened to the audio of each interview twice. The transcripts were then coded based on the direct information that was provided within each individual interview. These codes were reviewed by GH and the other Autistic members of the research team (HC, TM and SG) and the data was coded again prior to the themes being identified. This process was iterative, and the themes were discussed and refined over multiple meetings with the research team. The themes were then developed into research priorities based on their shared meaning. This was done separately for Autistic adults and Autistic young people. Eleven draft research priorities were identified for Autistic women and gender diverse adults, including subtopics under each of these priority areas (total of 65 subtopics). Six draft research priority areas were identified for Autistic girls and gender diverse young people, including 28 subtopics. These draft research priority areas are outlined in Table 1.

**Table 1:** Draft research priority areas for Autistic girls, women and gender diverse people

Autistic girls and gender diverse young people		Autistic women and gender diverse adults	
1.	Being accepted and understood	1.	Support in adulthood
2.	Challenges of being an Autistic girl or gender diverse person	2.	Knowledge about the experiences of Autistic women and gender diverse people
3.	Understanding and education about autism in girls or gender diverse people	3.	Mental health
4.	Experiences of school, friendships and relationships	4.	Experience of Autistic trauma
5.	How to provide support for Autistic girls and gender diverse people	5.	Advocacy and education
6.	How you are expected to act as an Autistic girl or gender diverse person	6.	Harnessing Autistic strengths and passions
		7.	Risk of sexual violence or abuse
		8.	Experiences of discrimination
		9.	Autistic community and culture
		10.	Physical health
		11.	Experience of gender and gender norms

**Fig. 1:**
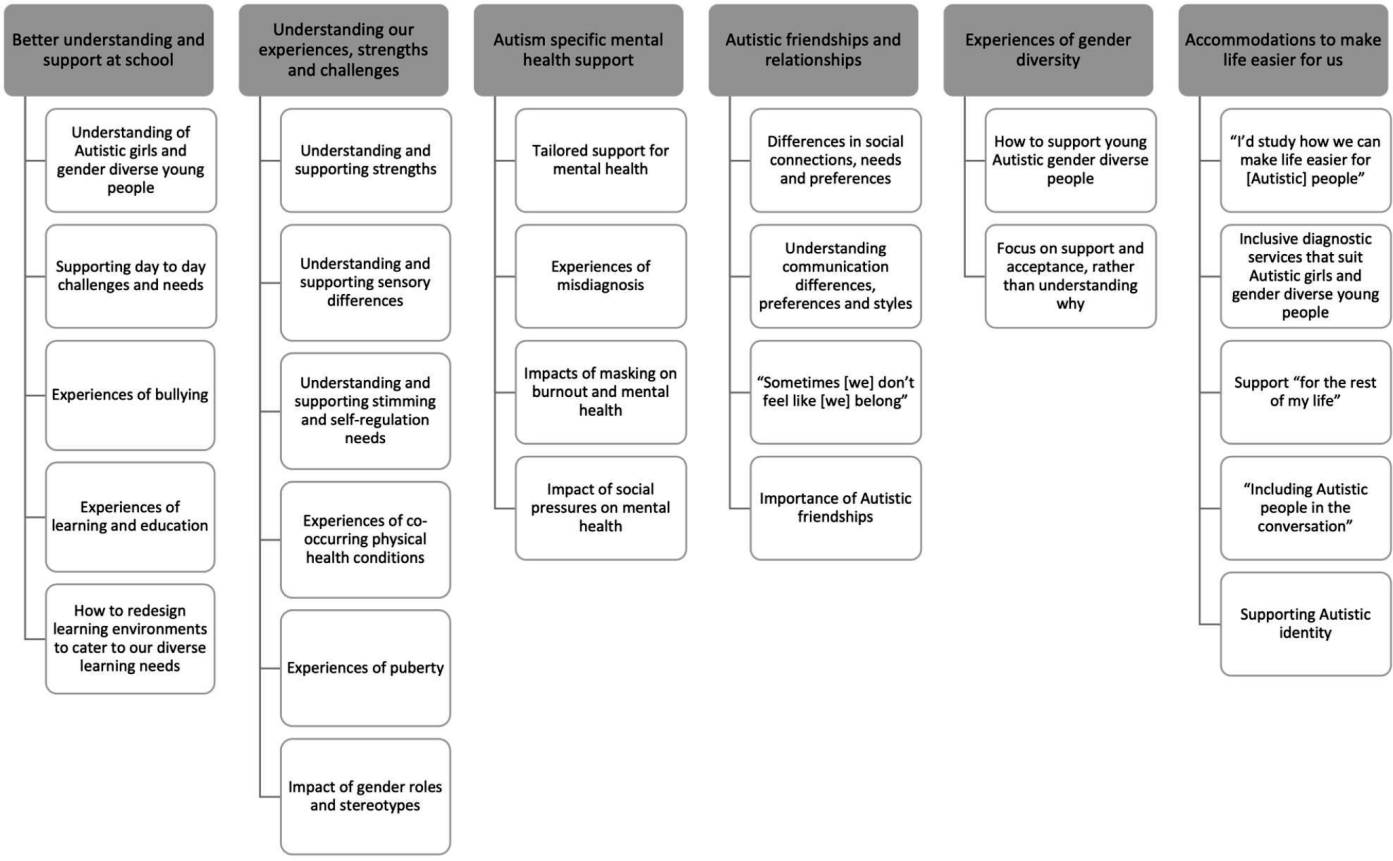
Research priorities for Autistic girls and gender diverse young people

**Fig. 2:**
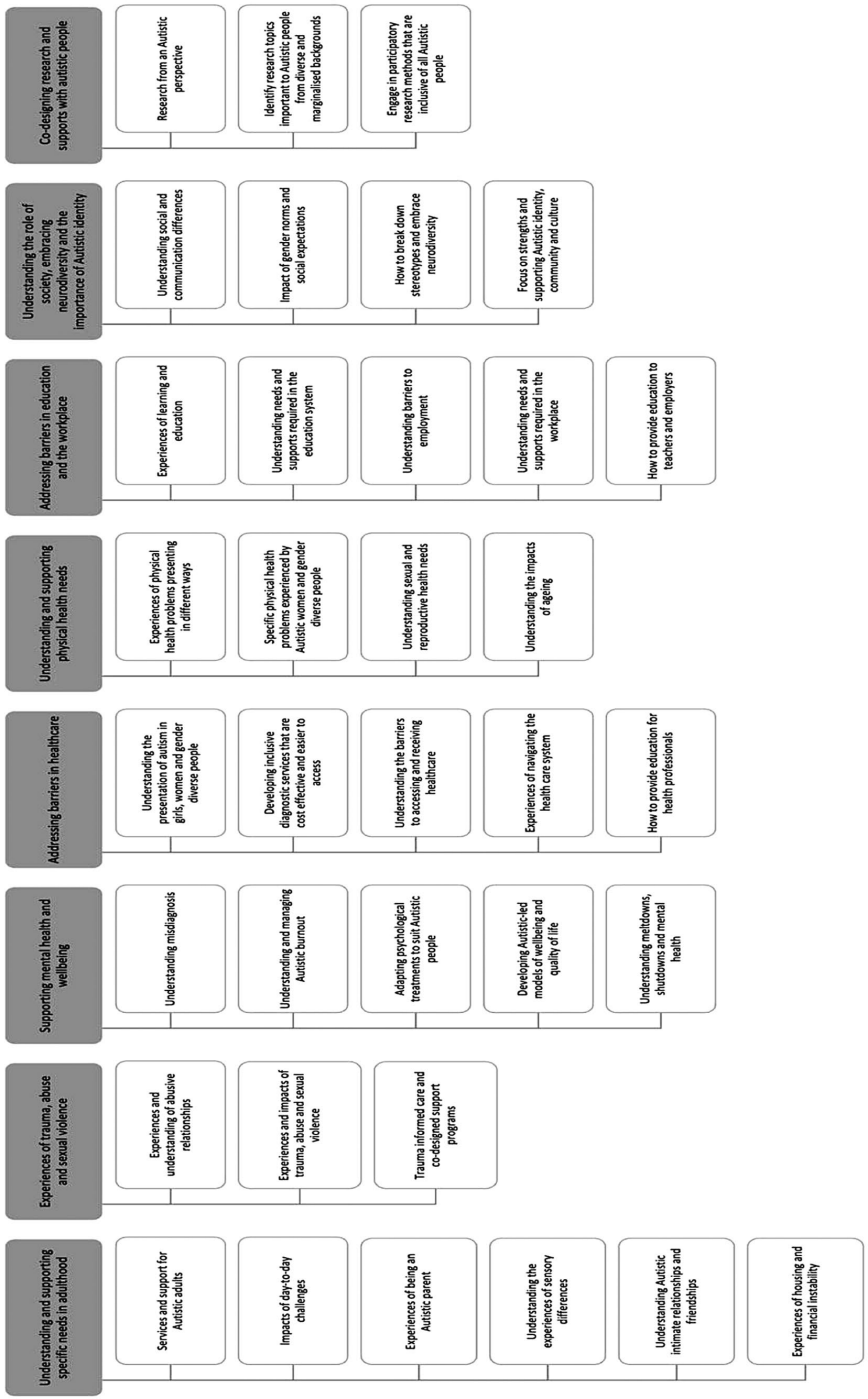
Research priorities for Autistic women and gender diverse adults

The online survey data for Autistic adults was ranked from one to eleven, with the subtopics under each priority area ranked according to how often they were selected as one of the top three subtopics. Following this, the research team discussed any areas where the research priority areas and subtopics overlapped, and these were combined. While completing this process, it was ensured that the top three priorities under each draft priority area were retained within the final set of research priorities for Autistic women and gender diverse people. The additional comments provided by the Autistic adults who responded to the survey were also reviewed to identify any additional areas of research that were not included in the initial draft priority areas. These were discussed by the research team to determine if there was sufficient consensus to include them, and those identified were then included as subtopics where appropriate. A similar process was followed for the online survey data for Autistic girls and gender diverse young people. However, this was also undertaken in consultation with an Autistic young person, who assisted with finalising the research priority areas for Autistic young people. This process resulted in six final research priorities for Autistic girls and gender diverse young people, including 26 subtopics (see Fig. 1). Eight priority areas were identified for Autistic women and gender diverse adults, including 35 subtopics (see Fig. 2).

## Results

Table 2 provides demographic information for the interview and survey data. Additional details about co-occurring physical and mental health conditions are provided in Table 3.

**Table 2:** Demographic information

	Autistic young people		Autistic adults	
	Interview (*n* = 16)	Survey (*n* = 81)	Interview (*n* = 31)	Survey (*n* = 330)
	%	%	%	%
**Gender identity**				
Female	81.3	63.0	83.9	80.9
Non-binary	18.8	24.7	6.5	13.4
Transgender		2.5	3.2	1.8
Gender fluid			3.2	0.9
Gender queer		3.7		0.9
Other		2.4	3.2	1.5
Prefer not to say		3.7		0.6
**Ethnicity**				
Aboriginal or Torres Strait Islander		4.9	3.3	3.3
White European	93.8	71.6	76.7	86.1
White other	6.3	18.5	10.0	9.1
South Asian / South East Asian	6.3	6.2	3.3	4.8
Other		2.4	6.7	7.3
**Autistic parent**			40.0	47.8
**Non-speaking**		4.9		0.6

**Table 3:** Co-occurring mental health and physical health conditions

	Autistic young people		Autistic adults	
	Interview (*n* = 16)	Survey (*n* = 81)	Interview (*n* = 30)	Survey (*n* = 330)
	%	%	%	%
**Neurodivergence**				
Intellectual Disability	12.5	1.2		2.4
Attention Deficit Hyperactivity Disorder (ADHD)	50.0	50.6	26.7	44.5
Dyslexia	6.3	18.5	10.0	7.0
Dyspraxia or Developmental Coordination Disorder	6.3	4.9	3.3	4.5
Sensory Processing Disorder		7.4	3.3	
Other	6.3	7.4	13.4	5.1
**Mental health**				
Anxiety disorder	75.0	76.5	53.3	81.5
Bipolar disorder		2.5	10.0	9.7
Obsessive compulsive disorder	18.8	18.5	10.0	14.8
Post-traumatic stress disorder (PTSD) / Complex PTSD		12.3	40.0	36.4
Depression	31.3	34.6	40.0	75.5
Drug or alcohol misuse		2.5	3.3	13.0
Dissociative Identity Disorder			3.3	0.6
Eating disorder		16.0	16.7	23.3
Borderline Personality Disorder		3.7	3.3	8.7
Personality disorder (other)			10.0	1.2
Schizophrenia		1.2		1.8
Gender dysphoria	6.3			
Other		9.9		4.2
**Physical health**				
Chronic fatigue syndrome		7.4	6.7	16.7
Chronic pain		12.3	26.7	23.3
Epilepsy		1.2	3.3	1.5
Ehlers Danlos syndrome		7.4	3.3	7.6
Fibromyalgia			6.7	0.9
Gastrointestinal issues	6.3	17.3	50.0	35.8
Hypermobility Spectrum Disorder	6.3	11.1		10.9
Immune conditions			10.0	0.6
Pernicious anaemia			3.3	0.3
Polycystic ovarian syndrome			13.3	0.3
Sleep issues	50.0	2.4	26.7	
Thyroid problems			10.0	
Other			6.7	4.8

### Research Priorities for Autistic Girls and Gender Diverse Young People

Autistic girls and gender diverse young people identified six key research priority areas that would benefit their lives. These are outlined in Fig. 1.

#### Better Understanding and Support at School

The first research priority area identified by Autistic girls and gender diverse young people is related to the need for an increased understanding of their learning experiences, including “that I learn differently to all the other students” (YP14). Autistic young people also spoke about the challenges that arise from being in an environment that often is not responsive, accepting or accommodating to their diverse learning needs:

Schools… should improve a bit on how much the teachers know about autism in general and neurodivergence, ADHD, all these different things. Because it’s very common and schools aren’t always set up to help these kids. I think there could be quite a lot of improvement in the education system (YP7).

There is also a need for teaching staff to respond to the needs of their Autistic students, particularly related to stimming and self-regulation. This includes understanding the impact of sensory sensitives in the classroom related to hearing “all the different conversations all at once and it’s highly overwhelming and you can’t focus in on one that you might need to be listening to” (YP16).

Additional research subtopics included understanding experiences of bullying, as well as research focused on how to provide a better understanding of Autistic girls and gender diverse young people in the education system:

The fact that I can be in a school system where I don’t receive the support I need because I’m not visibly struggling. I think there needs to be more awareness spread of how autism looks and that it’s not a little mould that someone can fit into (YP16).

#### Understanding our Experiences, Strengths and Challenges

The second research priority area outlines the need to understand and support both the strengths and challenges experienced by Autistic girls and gender diverse young people. This includes research to better understand experiences of puberty and what it is “going to actually do to the rest of my life” (YP2), as well as “the physical conditions that come along with [being Autistic]” (YP4). There is also a need for research focused on understanding and supporting sensory differences, self-regulation needs and “that stimming and stuff makes me happy” (YP6). The impact of gender roles and stereotypes on Autistic girls and gender diverse young people was also noted to be important:

I was a bit confused about all this stuff because I didn’t really see representation of an Autistic person that looked or acted like me, a girl, a teenager, all of these things (YP7).

Autistic girls and gender diverse young people also highlighted their incredible strengths, including “my ability to accept people for who they are. I think I’m a very genuine person and I can see people… what they might be struggling with and what strength they might have” (YP16) and “I’m very funny and I like helping people” (YP8). This highlighted the importance of recognising strengths such as compassion and wanting to help others, alongside the challenges experienced by Autistic girls and gender diverse young people.

#### Autism Specific Mental Health Support

Autistic girls and gender diverse young people identified the need for research focused on how to better support their mental health. This includes further understanding their experiences of misdiagnosis and the impact of masking:

Our desire to fit that mould means we do mask a lot of ourselves and we do hide who we truly are to try and fit that. I think becoming yourself is a big journey that takes a lot of work and a lot of self-acceptance that we so badly want to fit that neurotypical mould that when we realise that we can’t, there’s a lot of mental juggling that we have to do (YP16).

Autistic girls and gender diverse young people also outlined the need for research to understand their experiences of burnout and the impacts of “constantly trying to fit in with everyone and having no idea, and I would have massive burnouts and that just depleted me” (YP9). Mental health was also impacted by society through “a pattern of Autistic people, girls in particular, crashing in their early teens... because of having to deal with increased social pressures” (YP7). Autistic girls and gender diverse young people are asking for critical research into how to adapt mental health supports to meet their needs.

#### Autistic Friendships and Relationships

The fourth research priority area focuses on the need to better understand social experiences, connections and relationships. This includes understanding how “I can get very stressed with too much social stuff, which is hard because I’m actually a very social person… If I spend a lot of time with people I have to take a few days to recover” (YP7). Autistic girls and gender diverse young people also identified the need to understand their communication differences such as finding it “difficult to know when to reply and when not to speak” (YP5) and experiences of feeling “like [we] don’t belong” (YP3). This priority area also highlighted the importance of Autistic friendships and connections with other Autistic people and how “thinking that I wasn’t the only one… made me feel a lot better” (YP8).

#### Experiences of Gender Diversity

There is a need for research that focuses on understanding “the relationship between autism and gender diversity and the challenges of an Autistic person in understanding gender” (YPS4). Autistic girls and gender diverse young people emphasised the importance of approaching this research from a place of acceptance of gender diversity, with the need to “focus on support first” (YP7), rather than trying to understand why Autistic people may not identify with their gender assigned at birth or the gender binary. It is critical that future research is conducted in partnership with young Autistic gender diverse people, to ensure that research priorities or recommendations for supporting Autistic gender diverse young people are inclusive and appropriate.

#### Accommodations to Make Life Easier for Us

The final research priority area identified the need for research focused on “how we can make life easier” (YP13) for Autistic young people. This was related to both research and advocacy, and the need for “charities focused on actual support for Autistic people” (YP7). Autistic girls and gender diverse young people also highlighted the need for the re-evaluation of the current diagnostic process to ensure they are identified earlier. As one young person told us:

I spent years struggling in school until I mentally broke. I think this is an issue that needs to be addressed. Why does it take so long to help Autistic females? Why do we have to be mentally exhausted? (YPS6).

Autistic girls and gender diverse young people also identified the importance of challenging how society views autism and the need for acceptance from broader society, but also within research, as “a lot of people misunderstand [us]… even researchers usually misunderstand [us] and treat [us] like babies” (YP4). Research focused on “more regional” (YP1) access, support “for the rest of my life” and concerns that “when I get older, how is it going to change their reaction to me being Autistic” (YP2) is also needed. Autistic girls and gender diverse young people also highlighted the importance of “including Autistic people in the conversation” (YP7) when it comes to research, and the importance of Autistic identity as “something that you should be teaching to other Autistic girls, that it’s nothing to be ashamed of. If anything, it’s something to be proud of” (YP12).

### Research Priorities for Autistic Women and Gender Diverse Adults

Figure 2 outlines the final research priority areas identified by Autistic women and gender diverse people.

#### Understanding and Supporting Specific Needs in Adulthood

The first research priority area identified the need for understanding autism across the lifespan. The Autistic women and gender diverse people we spoke to reported that the accommodations provided are based “around children… there’s nothing for adults” (AD30) and that “it’s like once you’re out of nappies no one’s interested…No one wants to know about an Autistic adult” (AD15). Autistic women and gender diverse people also identified the need for research to understand the impact of day-to-day challenges such as “adulting… cooking, cleaning, organising paying the bills” (AD2) and for this to be linked to funding and support. Research also needs to focus on understanding the sensory experiences of Autistic women and gender diverse people, including how “women are more sensory orientated, that we have more sensory needs than men” (AD14), and the impacts of “taking all [the] senses in” (AD11). It is also important to consider the “sensory aspect” of “having my period each month” (AD2) and the need for funding bodies to accommodate the need for “simple things like sensory clothing; you can get underpants and socks, but you can’t buy bras” (AD30).

Additional subtopics identified under this priority area were related to understanding intimate relationships and friendships, including the importance of “having someone I can be safe with” (AD25) and “how you actually participate in a physical sexual relationship if you have lots of sensory issues” (AD1). There is also a significant lack of research focused on Autistic adult’s experiences of housing and financial instability. One Autistic adult spoke about their experience of being in an assisted living facility and how “this space that I’m in I’m made to feel like it’s not mine and how I choose to have it is not okay” (AD5). Finally, Autistic women and gender diverse people identified the need for research to understand their experiences of being a parent, as “everything around parenting and autism seems to be focused on parents of [Autistic] children, not [Autistic] parents” (AD20).

#### Experiences of Trauma, Abuse and Sexual Violence

The second research priority area identified by Autistic women and gender diverse people highlights the importance of understanding the experiences and impacts of being “forced into these situations that are traumatic” (AD16) and “how to avoid the continual pattern of repeated trauma in Autistic women’s lives” (AD19). This includes research on sexual violence, to understand their experiences of being “sexually assaulted by someone I thought I trusted” (AD27), “instances of assault from my male peers” (AD4) and that “teenage girls that are Autistic would be highly susceptible to being taken advantage of” (AD30). There is also a need to understand abusive relationships and the impacts of being “financially abused, I was emotionally abused. I was gaslighted” (AD22). Abuse and trauma need to be considered not only within interpersonal relationships, but also within systems and society, to better understand the supports that Autistic women and gender diverse people need. Some examples provided within this study were related to the child protection system and being “scared of having my daughter taken away from me” (AD8) and accessing funding for support:

How do you prove child abuse? How do you prove spousal abuse, when nobody believed you at the time?… The whole system is intrusive and abusive (AD22).

The impacts of “abuse of various types combined with autism caused incredible trauma. Complex repetitive trauma” (AD10) highlights the urgent need for research focused on how to prevent violence against Autistic women and gender diverse people and provide trauma informed care and support. This is critical for gender diverse Autistic people, who experience higher rates of discrimination and gender-based violence.

#### Supporting Mental Health and Wellbeing

Autistic women and gender diverse people identified the need for research that focuses on supporting their mental health and wellbeing. It is important to understand mental health and wellbeing within the context of gender roles and neuro-normative expectations in society, and “feeling that you have to really fit in… a square hole when you’re a round peg” (AD9). The Autistic women and gender diverse people we spoke to described the significant impacts of fitting with these societal expectations on their mental health, including on meltdowns and shutdowns, and the need for these to be better understood:

It’s a nightmare. It’s constant stress. I have a meltdown nearly every single day (AD12).

This was also related to “a lot of… burnout moments” and “a few stints in the mental health system because of that” (AD26). These experiences also highlighted the need for research to understand the impacts of misdiagnosis of “personality disorders. Things that I felt didn’t really fit” (AD13), that were “completely just wrong” (AD10) and being “placed through a lot of trauma… saying I was depressed, I was this” (AD28). This was sometimes associated with receiving harmful care such as being “heavily… inappropriately drugged” (AD10), as well as inappropriate treatment that failed to consider the impact of sensory differences:

I have had years of [Cognitive Behaviour Therapy]… all that sort of therapy has never worked. And they didn’t take into account when I mentioned, the lights are so bright in the shops and the noise is too much… I was always told that was anxiety. No, it’s not anxiety, it’s actually sensory overload (AD13).

Autistic women and gender diverse people are calling for research that focuses on co-designing therapeutic supports and for current therapeutic approaches to be adapted to meet their needs. This is critical to ensure that Autistic people are not “stuck for 20 odd years in the mental health system. Because it breaks you” (AD13). There is also a need to develop Autistic-led and co-produced models of wellbeing to understand “how to help Autistic people have a good life on their own terms” (AD7). Research also needs to consider the experiences and needs of Autistic women and gender diverse people with high support needs who “do not have the privilege of being able to mask my autism” (ADS1). These differing experiences need to be incorporated into research and support programs for Autistic adults.

#### Addressing Barriers in Healthcare

Further research is needed to identify and address the barriers and health inequities experienced by Autistic women and gender diverse people within the health care system. This includes understanding their experiences of navigating, accessing and receiving medical care. There is a significant need to increase health professionals’ understanding of autism, to meet the needs of their Autistic patients and improve the quality of and provision of healthcare. This includes understanding the presentation of autism in girls, women and gender diverse people, to ensure “that the GP is going to know about autism in women and not just think that it’s a thing for little boys with behavioural problems” (AD29). There is also a need for research into how to provide education for health professionals. As one Autistic adult stated:

They are not seeing it, they are not taught it. I don’t believe they have the education… I feel that’s a big area that needs somehow needs changing (AD13).

Autistic women and gender diverse people identified the need for research that addresses the barriers they experience being a “woman with a disability trying to navigate the healthcare system” (AD17) including “dealing with doctors and going into hospital… bright lights, people running in and out” (AD16). Others described the need to address the impacts of the “patriarchal nature of medicine” (AD17), as well as common experiences of gaslighting and not feeling understood or supported by healthcare professionals. As one autistic adult described:

I’ve had these diseases for nine years now… That’s nine years of the same specialist. And really it’s only been in the last couple of years that he’s starting to listen to some of the things that I say (AD15).

Research needs to then identify how to tackle these barriers, not only at the individual or interpersonal level, but from a whole system perspective through making changes within society and policy. This includes addressing additional barriers such as “being regional. The local area doesn’t seem to have the resources, medically, mental health wise or anything, to support people” (AD22). It is also critical to understand the healthcare experiences of Autistic people with high support needs who “have been denied medical and mental health care because of my level of autism” (ADS1).

#### Understanding and Supporting Physical Health Needs

In addition to addressing these barriers in healthcare, Autistic women and gender diverse people also identified the need for research that focuses on recognising and supporting their physical health needs. This includes a better understanding of their experiences of ageing and “how as I age my body is going to change and I don’t know what’s going to happen because there is no long-term research” (AD28). Research also needs to focus on specific experiences of physical health such as having different “presentations in my diseases… the textbooks don’t agree with” (AD15), as well as the impacts of having multiple chronic health conditions; “if I start reeling off my health problems, I would need another 45 minutes, and that’s if I just name the conditions” (AD12). It is critical for research focused on the specific physical health conditions that Autistic women and gender diverse people may be more likely to experience, such as being “super hypermobile” (AD26) and having “chronic pain” (AD19). In addition, this priority area outlines the need for research focused on how to support the sexual and reproductive health needs of Autistic women and gender diverse people, including pregnancy and “childbirth as an Autistic person” (AD16). There is also a need to better understand the impact of “puberty and anything to do with menstruation” (AD10) and experiences of menopause; “we hit menopause… and our whole sensory system goes offline, and restructures itself” (AD14).

#### Addressing Barriers in Education and the Workplace

The sixth research priority area identified the need for research within the higher education system and the workplace to further understand the barriers and gaps that currently exist within these environments. Specific research areas include understanding experiences of learning and education and the impact this has on Autistic women and gender diverse people. As one Autistic adult described:

The education system is structured to fail these girls, to fail these women, and it goes on even after diagnosis… continually pigeoning us into these ideals of the way that we have to be… Constantly punished for being awesome, for being opiniated, for thinking outside the square, and actually having a very different view of the world (AD28).

Research into the support needs of Autistic women and gender diverse people within the education system, including experiences of not receiving “much support or understanding of anything, other than you can get an extension” (AD21) and the importance of providing teachers “with information about their students’ disabilities” so they can “better support their students” (AD2) is also required. The voices of Autistic people with high support needs also need to be included in this research, to address the harms that they often experience such as “seclusion and restraint in education settings” (ADS1).

Subtopics related to employment identified the need to better understand barriers to employment, including that “the interview process that you go through to get a job is not Autistic-friendly at all” (AD30). Others also described stereotypes as an additional barrier to employment, including how some employers “commonly advertise [for] a very specific kind of Autistic person”:

It always seems to be the very data, analytical, puzzle loving guy… advertising, researching and understanding only those sort of Autistic people, is leaving behind all kinds of people who aren’t like that… I end up feeling like, if I’m not that kind of person, is there any upside to my own ‘autism’ within a job? I also wonder how many other girls out there feel the same way (AD21).

Autistic women and gender diverse people are calling for research into how best to provide education to both teachers and employers. This is particularly relevant not only for new teachers, but also for “educating teachers or people of a generation who are about sixty and up… or people who have that cookie-cutter mindset of what autism is” (AD18). There is also a need for research into how to make learning environments and workplaces more accessible and supportive for Autistic women and gender diverse people. As one Autistic adult stated:

The infrastructure isn’t there or the education isn’t there… It really is a massive issue of equality and equity, and I feel like I’m always just hanging onto my job, and the only way I can do it is to play the part (AD9).

#### Understanding the Role of Society, Embracing Neurodiversity and the Importance of Autistic Identity

Autistic women and gender diverse people identified the need for research related to providing a better understanding of the role of society in shaping their experiences:

I think when it comes to Autistic girls, the message is given to us… you have to be polite. You have to do this and hug your uncle… do all the right social niceties. It’s a stronger message to girls (AD22).

This research priority also includes understanding the impacts of “adapting to meet other people’s needs” (AD14), the need for “more understanding of the differences in communication styles” (AD12) and social preferences of Autistic people and how “all of those social and sensory interactions take [their] toll” (AD1). Research into the intersection between gender roles and social expectations on masking and mental health is also critical. The Autistic women and gender diverse people we spoke to described feeling the need to mask their gender identity, “accumulated a very thick mask even once I knew that trans people were a thing” (AD5) and the impacts of having “spent my whole life masking” on “my mental health, and even my physical health” (AD19).

There is a need to embrace neurodiversity as the framework by which we celebrate, accept and support Autistic people and their way of being to provide “a broader understanding of the rich tapestry of Autistic people” (AD6). Research also needs to focus on “how you assist Autistic people [to] deconstruct a life that was constructed by neurotypicals. And then use a different framework to actually allow them to construct a life that’s actually based on their values” (AD6). Furthermore, there is a need to focus on the important role that Autistic identity, community and culture play in the wellbeing of Autistic people. The importance of fostering Autistic community was described by one Autistic adult:

I feel like that needs to be part of the diagnosis process… don’t teach them how to socialise with neurotypical people…Take them into groups of Autistic people where they can be themselves and be accepted and loved for who they are… Because this is your people (AD7).

Another Autistic adult spoke about the importance of recognising the strengths of Autistic young people and supporting them to flourish:

I think these girls, these strengths and these brains need to be embraced and utilised. I look at … these brilliant, brilliant human beings… I wished that I had those tools, and that I was allowed to thrive and to be who I am when I was that young (AD28).

#### Co-Designing Research and Supports with Autistic People

The final priority area identified by Autistic women and gender diverse people focuses on the need for Autistic people to be a fundamental part of the design process when it comes to autism research and supports. While the other seven research priorities indicate the topics that should be prioritised, this final research priority identifies the way in which this research should be conducted. Research “from the Autistic perspective” (AD14) is critical to develop research and support programs that are meaningful and relevant for Autistic women and gender diverse people. This research needs to be informed by the perspectives of all Autistic people, including non-speaking Autistic people, Autistic people with an intellectual disability and “Autistic women with very high support needs” (ADS3). It is also crucial for future research to identify research topics that are important to, and benefit, Autistic women and gender diverse people from diverse and marginalised backgrounds. The different experiences of Autistic people from migrant backgrounds was highlighted by one Autistic adult:

[Autistic] migrant women… are made to feel that any differences are due to race or culture… that’s one of the main reasons why I didn’t get diagnosed till I was 55…. Any differences, you tend to put this cultural interpretation… social difficulties are interpreted as cultural behaviour or lack of English (AD8).

## Discussion

This study aimed to develop key research areas that will benefit the lives of Autistic girls, women and gender diverse people in Australia. A number of the research priority areas correlate with some of those identified in previous research in Australia, including health and wellbeing, education, employment, service delivery, gender, diversity and inclusion (AARC, 2019, 2021). However, we identified the unique experiences, perspectives and needs of Autistic girls, women and gender diverse people within these priority areas. This included the impacts of specific factors such as gender roles, stereotypes of autism and neuro-normative expectations for Autistic girls, women and gender diverse people. These societal factors need to be considered within any research related to health and wellbeing, given their impact on burnout, misdiagnosis, mental and physical health. In addition, we identified the need to understand the specific physical health needs of this community, by identifying commonly occurring physical health conditions they experience, as well as how to support their sexual and reproductive health needs. There were also additional barriers identified specifically for Autistic women and gender diverse adults related to navigating the healthcare system, including not being understood by health professionals and incidents of gaslighting, or being made to question their own experiences or perceptions. Research in this area is particularly important for Autistic gender diverse people, who report more barriers to accessing gender-affirming care (Strauss et al., 2021).

There were also several additional priority areas that were identified by Autistic girls, women and gender diverse people in our study. Autistic adults identified the need for research focused on trauma, abuse and sexual violence. This includes understanding the risk factors for, and experiences of abuse and sexual violence within intimate partner and family relationships. While the current study did not focus on quantifying these experiences, our findings add to the emerging literature on Autistic people’s experiences of interpersonal and sexual violence (Cazalis et al., 2022; Gibbs et al., 2021; Gibbs & Pellicano, 2023) and outline a clear need for research in this area. According to the Australian Bureau of Statistics (ABS), approximately 11% of the Australian population are diagnosed with Posttraumatic Stress Disorder (PTSD) in their lifetime, increasing to 14% for women aged between 16 and 85 (ABS, 2020). However, our sample reported a substantially higher prevalence, with up to 40% of Autistic adults and 12% of young Autistic people reporting a diagnosis of PTSD. Gender diverse people also experience higher rates of verbal, physical, sexual and intimate partner violence than cisgender people (Callander et al., 2019; Hill et al., 2020; Yerke & DeFeo, 2016). These disparities underscore the importance of trauma-informed research and supports tailored for Autistic women and gender diverse people. Autistic women and gender diverse people also identified research priorities related to supporting their specific needs in adulthood, including day-to-day domestic tasks, the experiences and needs of Autistic parents, as well as research related to housing and financial instability. While these areas are under researched, there is some emerging literature focusing on Autistic parents (Gore et al., 2023; Heyworth et al., 2022), as well as experiences of homelessness amongst Autistic people (Garratt & Flaherty, 2021). Our findings highlight the need for additional research focused on how to support the specific needs of Autistic women and gender diverse people in these areas.

This is the first study to identify specific research priorities for Autistic girls and gender diverse young people. Our findings highlight that Autistic young people want to be part of the research that has an impact on their lives. Young people identified the need for research to support them within the education system, as well as to understand their physical and mental health needs. Gender roles, stereotypes and social pressures were also shown to significantly impact on Autistic girls and gender diverse young people, highlighting the need to consider the intersection of autism and gender identity within research and clinical practice. One of the key priority areas identified by Autistic girls and gender diverse young people was related to understanding the support needs of young gender diverse Autistic people. While this was identified by Autistic adults as important, this was highlighted as a critical area of need for Autistic young people in this study. This may be due to the increasing social awareness of gender diversity and legal protections for the rights of gender diverse people that young people are more likely to have been exposed to compared with the Autistic adults we interviewed. However, it also reflects the mismatch between the limited research in this area that often focuses on understanding the prevalence of Autistic people who may identify as gender diverse, rather than on their needs (Corbett et al., 2022). It is imperative for research focused on how to support Autistic gender diverse young people in healthcare, education and all facets of society. Working in partnership with Autistic gender diverse people is critical, to design research that is inclusive of this community.

There were several priority areas that were identified by both Autistic adults and young people. The first of these is related to mental health and wellbeing. This is not surprising, given the very high rates of mental health conditions reported by the Autistic girls, women and gender diverse people we spoke to, which ranged from 75 to 82% for Autistic young people and between 83 and 91% for Autistic adults. This is in stark contrast to the rates identified within the wider Australian population, with 45% of women aged between 16 and 85 (ABS, 2020) and approximately 13% of girls aged between 12 and 17 (Australian Institute of Health and Welfare, 2021) experiencing a mental health condition in their lifetime. In addition, young gender diverse Autistic people report higher rates of mental health conditions compared with non-autistic gender diverse people (Strauss et al., 2021), indicating the need for additional support. However, it is important to note that the high rates outlined in our study may also reflect the misdiagnosis noted by the Autistic girls, women and gender diverse people in this research, something which we did not specifically capture in this study. Autistic girls, women and gender diverse people outlined how traditional therapeutic supports had not worked well for them in the past, and the need for mental health supports to be adapted to meet their needs. They also described the need for research that focuses on how gender norms and expectations impact on masking, burnout and mental health. This is particularly important for gender diverse Autistic people, who experience additional stigma (Maroney & Horne, 2022) and higher rates of mental health conditions (Wallisch et al., 2023). Our findings highlight the need to provide education to health professionals to enable them to better support both the mental and physical health needs of Autistic girls, women and gender diverse people.

Autistic adults and young people also described their experiences of feeling misunderstood or mistreated by researchers and the importance of including “Autistic people in the conversation” (YP7) within research projects and teams. Both Autistic adults and young people identified clear priorities related to how autism research should be conducted, through the co-design of both research and supports with Autistic people. This is critical to ensure that research meets the needs of the Autistic community. Our findings are consistent with recent research conducted in New Zealand, that identified the importance of lived experience in research, as well as the need to include the perspectives of Autistic people with a diverse range of support needs (Emerson et al., 2023). We need to identify research areas that are important to Autistic people from diverse and marginalised backgrounds in Australia, as well as prioritise the perspectives of Aboriginal and Torres Strait Islander peoples, non-speaking Autistic people and those with intellectual disability and high support needs. To achieve this, we recommend including a diverse range of Autistic people as active members in research teams, to ensure that our research reflects the full diversity of the Autistic community in Australia.

Our research emphasises the importance of breaking down stereotypes and embracing neurodiversity both within research and in society, and supporting Autistic identity, community and culture to enhance wellbeing (Botha et al., 2022; Cooper et al., 2021; Ferenc et al., 2022). There is also a need to focus not only on the challenges experienced by Autistic girls, women and gender diverse people, but also on their strengths. We need to approach autism research using a gendered lens, socio-ecological model (Bronfenbrenner, 1989), underpinned by the neurodiversity paradigm (Pellicano & Houting, 2022) and social model of disability (Kim, 2021) to shift the focus from the individual, to understanding the interrelated influences of various factors at interpersonal, community and societal levels.

### Recommendations for Educators, Employers and Healthcare Professionals

There are valuable ways that the education, employment and healthcare sector can begin to make changes to better support the needs of Autistic girls, women and gender diverse people. This starts by listening to, learning from and sharing decision making power with Autistic people. Practical strategies include making an investment to ensure all staff have undertaken training in how to best support Autistic people within each of these sectors. Ideally this training should be neurodiversity-affirming, and developed and delivered by Autistic people. Healthcare services can make a commitment to establishing consumer groups for Autistic women, girls and gender diverse people, to provide valuable insight into how their practices and services can be more inclusive and accessible, including making environments sensory friendly and adapting communication techniques to capture the diversity of communication styles in the Autistic community. Mandatory training in trauma-informed practice is critical, given the prevalence of abuse and violence towards Autistic women, girls and gender diverse people. Work accommodation plans tailored to the needs of Autistic employees need to be provided to make the workplace more accessible, including support for flexible working arrangements. This also needs to be accompanied by sensory friendly workplaces, schools and tertiary institutions that meet the sensory needs of Autistic girls, women and gender diverse people. This includes quiet spaces for Autistic students to access whenever they need to. These spaces will not only support Autistic people, but also make these environments more accessible for everyone. In addition, it is critical that Autistic students have access to counsellors, psychologists and social workers who have experience in supporting the needs of Autistic girls, women and gender diverse people in an educational setting.

At an individual level, it is also important for researchers, educators, employers and healthcare professionals to think about how they can better engage with, listen to and learn from Autistic people. This might include attending professional development run by Autistic people, or reading literature written by Autistic authors. It is also important to engage in self-reflection to identify gaps in knowledge and areas where individual competence can be enhanced. This process of reflection and learning is ongoing, as no two Autistic people are the same and the needs of each individual Autistic student, employee or patient must be understood. Creating a safe space where Autistic girls, women and gender diverse people are supported to communicate, learn, interact and process in their own way is paramount. This means being open to doing things differently and being led by the Autistic person. It is imperative that all sectors commit to ongoing learning about autism, and more specifically the experiences of Autistic women, girls and gender diverse people. Removing stereotypical assumptions and stigma is foundational to ensuring that Autistic women and girls can flourish in employment, education and be better supported to access mental and physical healthcare.

## Limitations

This research includes the perspectives of Autistic girls, women and gender diverse people in Australia and may not reflect the research priorities of Autistic people worldwide, particularly in non-Western and lower and middle income countries. In addition, the people we spoke to were predominantly from a White European background, had received a diagnosis later in life, had a high level of education and were employed. While we captured a more diverse range of people in our survey, we need to ensure that we include the perspectives of all Autistic people in research, including Autistic people with intellectual disability, high support needs, complex communication needs and from marginalised backgrounds. Within this research project we were unable to reflect this diversity in the way that we wanted to from the outset, and left parts of the Autistic community feeling “invisible” (AS1). Without the inclusion of all Autistic people, we will remain firmly in our “echo chamber of privilege” (Reframing Autism, 2021) within autism research, only hearing the perspectives of the same select few. We as a research community need to look at how and why we are still failing Autistic people through not including their perspectives in our research. This includes ensuring that we correct the imbalance of power and shift the concept of ‘autism experts’ from researchers to Autistic people themselves. We also need to identify how research teams are doing this well, and be open to changing our research methods to ensure that they are accessible and inclusive, to capture the experiences and meet the needs of all Autistic people.

## Conclusions

This study highlighted key research areas for Autistic girls, women and gender diverse people in Australia. We are making a direct call to action by asking autism researchers to focus on these key priority areas and ensure that this research is Autistic-led or authentically co-produced (Grove et al., 2022). We need researchers to start listening to and working with Autistic people on the research priorities that are meaningful to them, and that will have a direct and positive impact throughout their lives. Focusing on the research priority areas identified in our study will lead to a better understanding of the experiences, perceptions and needs of Autistic girls, women and gender diverse people in Australia, which will positively impact their health and wellbeing, quality of life, and access to supports and services.

## Data Availability

Given the sensitive nature of the information contained in this data, it is not available to be shared.

## Footnotes

1 We refer to the Autistic and autism communities in this publication The Autistic community is made up of Autistic people. The autism community includes non-autistic people who are connected to Autistic people in various ways, including as allies, family members and people who support or regularly work with Autistic people. We also capitalise the word Autistic in this publication, to acknowledge the culture and community associated with Autistic identity. This is similar to the use of capitalisation within the Deaf community.
